# Lipid Metabolite Biomarkers in Cardiovascular Disease: Discovery and Biomechanism Translation from Human Studies

**DOI:** 10.3390/metabo11090621

**Published:** 2021-09-14

**Authors:** Peter McGranaghan, Jennifer A. Kirwan, Mariel A. Garcia-Rivera, Burkert Pieske, Frank Edelmann, Florian Blaschke, Sandeep Appunni, Anshul Saxena, Muni Rubens, Emir Veledar, Tobias Daniel Trippel

**Affiliations:** 1 Department of Internal Medicine and Cardiology, Charité Campus Virchow-Klinikum, 13353 Berlin, Germany; peter.mcgranaghan@charite.de (P.M.); burkert.pieske@charite.de (B.P.); frank.edelmann@charite.de (F.E.); florian.blaschke@charite.de (F.B.); 2 Baptist Health South Florida, Miami, FL 33143, USA; AnshulS@baptisthealth.net (A.S.); MuniR@baptisthealth.net (M.R.); EmirV@baptisthealth.net (E.V.); 3 Metabolomics Platform, Berlin Institute of Health at Charité Universitätsmedizin Berlin, 13353 Berlin, Germany; jennifer.kirwan@bih-charite.de (J.A.K.); MarielAnel.GarciaRivera@mdc-berlin.de (M.A.G.-R.); 4 Max Delbrück Center for Molecular Research, 13125 Berlin, Germany; 5 School of Veterinary Medicine and Science, University of Nottingham, Leicestershire LE12 5RD, UK; 6 DZHK (German Centre for Cardiovascular Research), 13353 Berlin, Germany; 7 Berlin Institute of Health, 13353 Berlin, Germany; 8 German Heart Center Berlin, Department of Cardiology, 13353 Berlin, Germany; 9 Department of Biochemistry, Government Medical College, Kozhikode, Kerala 673008, India; sandeepappunni@gmail.com; 10 Department of Biostatistics, Florida International University, Miami, FL 33199, USA; 11 Division of Cardiology, Emory University School of Medicine, Atlanta, GA 30322, USA

**Keywords:** metabolomics, lipidomics, biomarkers, cardiovascular disease, heart failure

## Abstract

Lipids represent a valuable target for metabolomic studies since altered lipid metabolism is known to drive the pathological changes in cardiovascular disease (CVD). Metabolomic technologies give us the ability to measure thousands of metabolites providing us with a metabolic fingerprint of individual patients. Metabolomic studies in humans have supported previous findings into the pathomechanisms of CVD, namely atherosclerosis, apoptosis, inflammation, oxidative stress, and insulin resistance. The most widely studied classes of lipid metabolite biomarkers in CVD are phospholipids, sphingolipids/ceramides, glycolipids, cholesterol esters, fatty acids, and acylcarnitines. Technological advancements have enabled novel strategies to discover individual biomarkers or panels that may aid in the diagnosis and prognosis of CVD, with sphingolipids/ceramides as the most promising class of biomarkers thus far. In this review, application of metabolomic profiling for biomarker discovery to aid in the diagnosis and prognosis of CVD as well as metabolic abnormalities in CVD will be discussed with particular emphasis on lipid metabolites.

## 1. Introduction

Cardiovascular disease (CVD) is the leading cause of death worldwide, accounting for 17.8 million deaths per year and its incidence rates are rising [1]. Dysfunctional cardiac energy metabolism is a major contributor to CVD. Specifically, lipids are of central importance for the bioenergetic metabolism of the heart and are the primary focus of CVD metabolic research. It is known that in the healthy heart, fatty acids (FAs) account for 60–90% of ATP production while glucose provides the remainder [2]. The failing heart shifts away from lipids toward a greater reliance on glycolysis, ketone body oxidation, amino acids (e.g., branched-chain amino acids or BCAA), and lactate as sources of energy [3]. Despite the many advancements in our understanding of cardiac metabolism, elucidation of these metabolic pathways remains a challenge, as well as the translation of metabolic findings to the clinical setting, such as improved or novel diagnostic/prognostic biomarkers.

**Figure 1:**
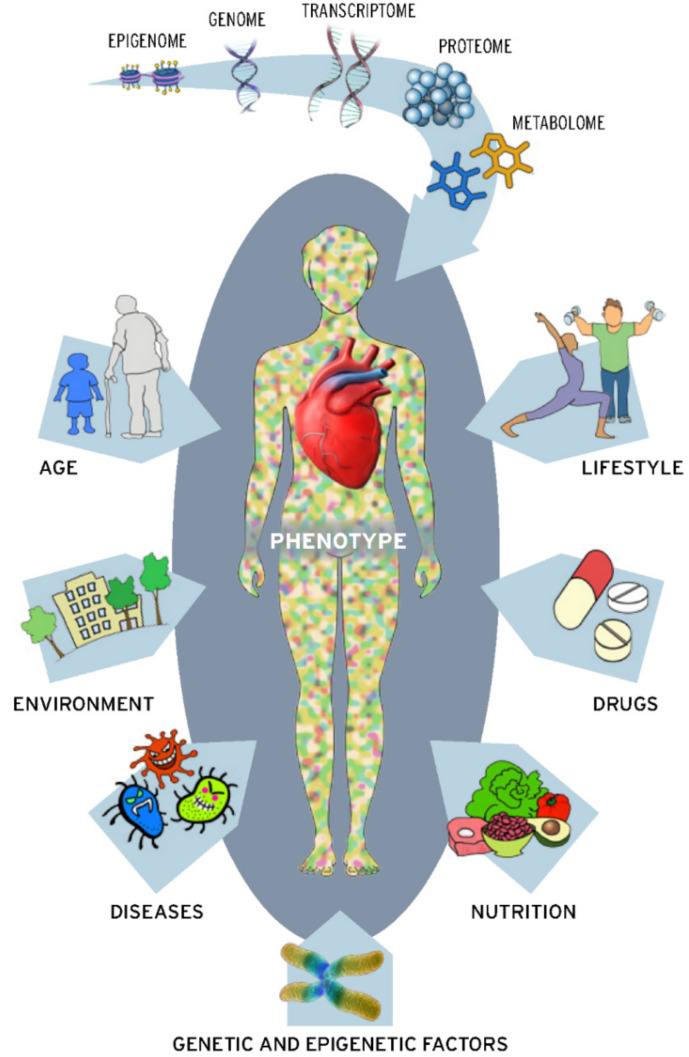
Overview of the -omic hieararchy and contributing factors to an individual’s phenotype.

Metabolite profiling, or metabolomics, is the latest -omics approach for characterizing small-molecule metabolite intermediates from canonical biochemical pathways and may be a useful technology for dissecting biomarkers and mechanisms of metabolic dysfunction in CVD. A metabolomic biomarker is different from a genomic, transcriptomic or protein biomarker, since metabolomic biomarkers typically comprise of groups of co-related metabolites that change in concert, rather than the more independent changes observed from other -omics biomarkers. The interdependence of metabolites results in a disease signature which can be used to more precisely identify or predict disease states. It is ultimately the metabolome, which provides biochemical feedback across all-omics layers, which represents the closest link to the phenotype (Figure 1).

The development of the lipidomics field, a subset of metabolomics which analyzes lipid metabolites and related biochemical pathways, is particularly relevant as lipids have previously shown to play a key role in the pathophysiology of CVD. Lipidomic techniques use various methods such as nuclear magnetic resonance spectroscopy (NMR) and mass spectrometry (MS) which can measure hundreds or even thousands of different lipid metabolites. Despite the significant advantages of metabolomic biomarkers, no diagnostic tests based on metabolomics have been introduced for clinical use; however, several metabolomic prognostic biomarkers have been validated and one is recommended by the Mayo Clinic (https://news.mayocliniclabs.com/ceramides-miheart/. Accessed 5 June 2021). Several challenges could explain this lack of clinically useful metabolomic biomarkers in cardiology practice. Challenges such as designing prospective metabolomic studies with sufficiently powered and representative cohorts, availability or cost of sample analysis using the proper technical platforms, analysis of complex metabolomic and clinical datasets with effective knowledge translation to identify candidate biomarkers, and finally, external validation of putative biomarkers.

In this review we highlight these challenges as well as different approaches for the development of lipid metabolite biomarkers, summarize the findings of recent lipidomic studies in human CVD studies, and discuss and how these findings may or may not contribute to our understanding of the pathophysiology of CVD.

## 2. Lipidomic Biomarker Discovery Approach and Summarized Findings

We searched cohort-based studies which reported on circulating lipid-based metabolites and their association with CVD outcomes. Several layers of keyword search criteria were used in PubMed, Web of Science, and Google Scholar databases in the date range January 2010 to July 2021. The keywords ‘metabolomics’, ‘lipidomics,’ ‘lipid metabolite biomarkers,’ ‘cardiovascular disease,’ ‘heart failure,’ and their synonyms (i.e., fatty acids, metabolite profiling) were used. The results were filtered based on the following inclusion and exclusion criteria:

Inclusion criteria:

- Metabolomics studies using MS or NMR approaches
- Lipid or lipid-related metabolites selected in final model
- Human blood samples
- CVD outcomes
- Exclusion criteria:
- Exclusively proteomic or other non-metabolomic studies which did not incorporate MS or NMR approaches
- Non-lipid or lipid-related metabolites selected in the final model
- Meta-analyses or literature reviews
- Animal studies
- in vitro studies
- Non-CVD outcomes

**Figure 2:**
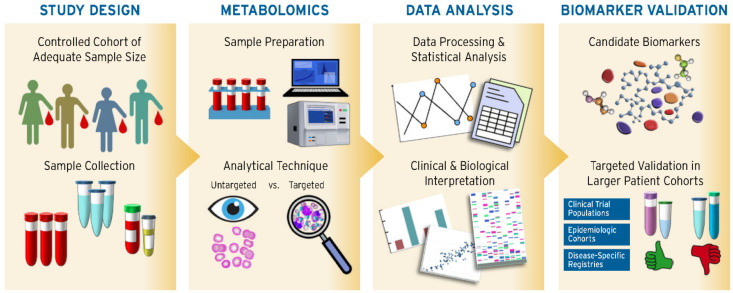
General metabolomic approach for biomarker analysis.

A total of 57 studies met these criteria and are summarized in Table 1. The designs of the studies, analytical techniques, data processing/analysis, biomarker validation, and translation to CVD pathomechanism are illustrated in Figure 2 and briefly discussed below. Some additional studies were included in the technical section as examples to demonstrate the use of particular methods.

### 2.1. Sample Selection

Metabolomic studies can be performed on a variety of biological matrices, including serum, plasma, urine, cerebrospinal fluid, tissue extracts [62], and even on stool samples [63]. For CVD biomarker studies, plasma or serum is typically used since blood-based biomarker tests are minimally invasive and the most practical to implement in the clinic. Serum is devoid of clotting factors and has both a different metabolite profile and different concentrations of individual metabolites than plasma, which is the cell free component of blood and has been treated with an anticoagulant [64]. Plasma is more commonly used in CVD metabolomic studies because of its quicker and simpler processing, better reproducibility, and the lack of time-consuming and potentially variable clotting process [65]. When preparing the sample, there is potential for introducing confounding factors if blood samples are collected and processed without proper standardized operating procedures (e.g., fasting, standardized time and processing procedure of blood, same brand, batch and type of anti-coagulant tubes). Where possible, confounding factors should be controlled for as part of the design-of-experiment (DoE) including the type of anti-coagulant used, hemolysis, excessive freeze-thaw cycles, storage time and excessive room temperature exposure, or matching potential clinical confounding factors between groups (e.g., age, sex, BMI, medication, smoking status, location) [66]. Specific metabolite biomarkers can also be used to control for sample quality, for example, to see whether blood plasma or serum has been appropriately collected and stored [67,68]. Some potential lipid biomarkers of disease e.g., O-phosphoethanolamines in serum, are also reported to change with only six hours storage at 4 °C [68]. Knowledge of such biomarkers enables certain samples to be flagged for removal on quality grounds.

In this review, the most commonly analyzed biological matrix was plasma (n = 43), and the next most common was serum (n = 17). There were 2 metabolomic studies which used both serum and plasma [17,47]. Sample selection and storage specifications were not consistently reported across the studies. In some cases, samples were stored for 15 years or more prior to analysis [6,11,12,54] in which degradation of some metabolites is possible. We are not aware of any of the studies from our review that reported on the measurement of preanalytical quality markers or adjusted for sample preanalytical confounders. Most studies (n = 37) included a majority white or European population, and most studies (n = 35) included a majority male population. There was only one study which included an all-female population [36]. About half (n = 25) of the studies included a population with a mean/median age of 65 or older.

### 2.2. Untargeted and Targeted Approaches

Both NMR and MS platforms can be used to characterize metabolite profiles either in a targeted manner, or in an untargeted manner depending on the study design. Targeted metabolomics measures a distinct, well characterized set of metabolites of known identity—typically several dozen to hundreds. Mass spectrometers are more sensitive when operated in targeted mode, acquiring data only for ions with specific pre-determined mass-to-charge ratios (*m*/*z*) and their specific fragments. Although the targeted approach generates a narrower view of the metabolome that is biased toward a predefined set of analytes, researchers have more confidence in the output because they know the identity of the signals, it enables full validation of a method, and absolute quantification is possible.

By contrast, untargeted metabolomics attempts to analyze all metabolites within a sample in an unbiased manner mainly for hypothesis generating studies. In untargeted metabolite profiling, hundreds to thousands of signals are analyzed, of which the identities of most are unknown [69]. Although an untargeted approach can detect thousands of signals, it requires more time and resources in order to identify unknown metabolites and the *m/z* are often insufficient to confidently assign peak identities. Many detected signals are also not of biological interest or reproducible, leading to an excess of noise in the data. Some estimates put the number of molecular ion peaks to be as low as 5 to 10% of the final total of detected peaks. To aid in metabolite identification, spectral and retention time libraries of known analytical standards are typically used but many have typically focused on polar metabolites and have not been so useful for lipids. This is beginning to change. Available databases include ChemSpider (http://www.chemspider.com. Accessed 6 August 2021), METLIN [70], Human Metabolome DataBase (HMDB) [71], MassBank [72], mzCloud (https://www.mzcloud.org. Accessed 6 August 2021), GNPS (http://gnps.ucsd.edu/. Accessed 6 August 2021), LipidBlast [73], and NIST Mass Spectral Library (http://chemdata.nist.gov. Accessed 6 August 2021)). Identification has been further improved by the development of in-silico fragmentation tools for mass spectrometry data such as LipidFrag [74], LipiDex [75] and LipidMatch [76] which normally pair in-silico fragmentation with existing lipid databases to maximize coverage of lipid species annotation. For targeted applications, the focus is on desired analytes with pre-defined identities and external libraries are not required. The final selection of analytical platform for a CVD study will depend on cost and time requirements, targeted vs untargeted approach, and the identity of metabolites of interest.

In our review, there were n = 39 studies which used a targeted approach, n = 12 used untargeted, and n = 9 used both [9,24,26,27,30,31,59,60,61]. The number of lipids analyzed ranged between 3–400.

### 2.3. Analytical Platforms

Once the proper cohort, outcomes, targeting approach, and samples are prepared, a metabolomic analytical platform should be selected. In metabolomic biomarker studies, multiple platforms are ideally employed, such as MS or NMR, since no single analytical method can accommodate the entire metabolome. Certain analytical platforms are advantageous in some areas while they may also lack certain important capabilities. For instance, NMR requires fewer sample preparation steps and is non-destructive, so samples can be later used for further analysis. It is also highly reproducible. However, it is expensive, less sensitive than other methods and will not detect some of the less abundant compounds without further pre-analytical separation. Complex lipid mixtures also hold particular challenges for NMR due to their very similar structures. MS can be coupled to a variety of separation techniques including gas and liquid chromatography (GC and LC, respectively). MS is more sensitive and can detect a wider range of metabolites but is less reproducible compared to NMR. Although MS has been criticized to provide less information on chemical identity than NMR, MS fragmentation as well as recent advancements in bioinformatic methods for the automatic annotation of chromatographic-mass spectral data can provide some structural information [77,78,79,80]. The advantages and disadvantages of NMR and MS have been reviewed previously [66] and are briefly described below.

In lipidomics research the two main MS-based methods are LC-MS and shotgun lipidomics. Both rely on electrospray ionization (ESI) technology to convert a liquid sample into an ion spray which can then be directed into the mass spectrometer for analysis. Shotgun lipidomics is a technique which was prevalent at the beginning of CVD lipidomics research. This technique directly infuses an extracted and reconstituted sample into a mass spectrometer (e.g., direct infusion mass spectrometry, DIMS, or flow injection analysis, FIA) for the detection of lipid metabolites without any chromatographic or other forms of prior separation. By contrast, LC-MS uses liquid chromatography to separate the lipid species before they are analysed by the mass spectrometer. Shotgun lipidomics has the advantage that it can analyze hundreds of lipids with relative simplicity of operation and shorter run times than chromatographic techniques [81]. However, the lack of separation means there are larger matrix effects such as ion suppression or ion enhancement often caused by the alteration of ionization efficiency of target analytes in the presence of co-eluting compounds in the same matrix. One probable cause of ion suppression is a result of several species competing for charge in an electrospray droplet. This can decrease the detection capability and measurement accuracy of affected compounds [82]. Another matrix effect is ion enhancement, or an increase in ion efficiency [83]. Both matrix effects can dramatically affect sensitivity and quantitation, therefore, they must be evaluated when validating a lipidomic method. Correction for matrix effects can be performed by using a specialized internal standard calibration procedure, such as using a stable isotope-labelled (analogue) of the analyte as an internal standard. Other methods to correct for matrix effects have been previously reviewed [84]. Though both LC-MS and shotgun approaches can suffer from matrix effects, shotgun lipidomics is impacted more due to the lack of prior separation, resulting in a more complex lipid mixture entering the electrospray. For the same reason, shotgun lipidomics has a higher technical measurement variability between samples than other methods. Another considerable drawback for shotgun lipidomics as a method is the challenge of accurate identification of the various lipid species which share the same theoretical accurate mass. On the one hand, different strategies can be employed to mitigate for such shortcomings such as enhancing sensitivity by the derivatization of the amino head group [85,86,87]. On the other hand, depending on the method(s) used, differing amounts of structural information will be collected for each analyte. Quantification is also a challenge for isobaric/isomeric mass overlap between lipid species, which may or may not be from the same lipid class (e.g., the ammonium acetate adduct of a phosphatidylcholine ion [PC + HAc^−^]^−^ has the same mass as a negative phosphatidylethanolamine ion [PE + H^−^]^−^). Even where fragmentation is used to identify the head group, and thus the lipid class, some of the observed signal may still be due to another lipid species, for example, isobaric phosphatidlycholine species with different fatty acid chains and the same total number of double bonds (e.g., PC 16:0_22:6 and PC 18:2_20:4). Despite the potential drawbacks, shotgun approaches are still employed, including in some commercial kits (e.g., Biocrates GmBH, Innsbruck, Austria) which have reasonable success with this approach [88].

Having a separation step prior to mass spectrometry improves the identification, quantification accuracy and coverage of lipids. LC–MS is one of the most popular methods for lipidomic research because of the relatively low cost, quick turn-around, and high sensitivity. Due to its detection sensitivity, this platform can measure thousands of lipids from a very small sample volume. LC separates lipids based on their interaction with a stationary phase and into their respective physicochemical properties, i.e., carbon-chain length and the number of double bonds, all of which affect the retention time on the LC column. After chromatographic separation, the isolated lipids undergo ionization and frequently also a controlled fragmentation step or steps (MS/MS or MS^n^), and the molecular ions and/or their fragments are detected using a mass analyzer. LC-MS still suffers from matrix effects and ion suppression, but to a lesser extent than shotgun lipidomics.

NMR has increasingly been used in lipidomic studies. NMR exploits a property called “spin” that exists in nuclei types (including ^1^H, ^13^C, ^15^N, and ^31^P) [89]. A magnetic field briefly excites a specific nucleus type, and the resulting relaxation of the nuclei is then measured. The resonance frequency of each nucleus is influenced by the shielding of the magnetic field by its nearest neighbors and the couplings between nearby nuclear spins. It is these properties that enables NMR to give structural information. However, identification of individual lipid species in complex mixtures is more challenging and may require samples of the pure compound. In complex mixtures, the ^1^H NMR signals of other molecular species overlap in the same spectral regions as for lipids. In most CVD metabolomic studies it is important to classify individual species, since the relevant lipid classes are complex heterogenous groups of compounds with unique biological mechanisms [90]. Various methods have been developed to improve the identification e.g., 2D heteronuclear single quantum coherence (HSQC) [91,92], or aliphatic chain length by isotropic mixing (ALCHIM) [93] but with the loss of quantification ability, but most examples we give below have used variations of ^1^H NMR.

Using a separation method first, such as liquid chromatography (LC-NMR), could solve the main drawback of signal complexity and allow structural elucidation [94]. This technique has not gained much momentum in the field of CVD yet, although there are some pre-clinical examples [95]. Moreover, compared to LC–MS and shotgun lipidomics, NMR-based lipidomics is much less sensitive, requires relatively long measurement times and has a low sample throughput.

In our review we found that most studies (n = 53) used MS-based platforms; while shotgun lipidomics (i.e., FIA) was used in n = 6 studies, LC-MS was used in n = 36 studies and n = 9 used a combination of both. NMR was used in n = 4 studies, and n = 3 used both NMR and MS [57,60,61].

### 2.4. Data Processing and Analysis

Metabolomic analysis can generate hundreds to thousands of data points per sample. Thus, the ability to interpret and analyze metabolomics data relies heavily on advanced computational approaches. Considerations and challenges in metabolomics data processing and analyses have recently been reviewed [96,97]. Other reviews detail the step-by-step protocols for metabolomics data processing [98,99]. Appropriate processing of metabolomics data is essential to produce dependable and high-quality data sets that will ultimately be used for analyses, as briefly described below.

After samples are chemically analyzed, raw data are typically pre-processed using software from instrument companies or from open sources such as Skyline [100], XCMS [101], MZmine [102], Metaboanalyst [103] or NMRProcFlow [104]. The process is a little different for targeted and untargeted methods. The specificity of the data collected in targeted metabolomics makes data processing and analysis less labor intensive than larger more complex datasets of untargeted metabolomics. Crucially, it also allows for accurate absolute quantification in some instances. For MS data, this process identifies and quantifies features in the data (“peak picking”), aligns the same metabolic feature across different samples and normalizes data to account for technical differences. Chemical identification of the features may also take place at this point, and for targeted analysis, an absolute concentration can be ascribed if a calibration curve and an internal standard (ISTD) have been used. Data may also be corrected for inter and intra-batch drift, normally based on results from repeating analyses of quality control (QC) samples. After this correction, a quality assurance (QA) protocol is performed to remove metabolite features with poor repeatability across QC samples.

Once data is processed and ready for analysis, the association of individual metabolites with an outcome of interest is modeled using univariate and multivariate methods. Similar to issues in data processing, there currently exists little uniformity in the biostatistical analysis of metabolomics data. Where univariate methods are employed, false discovery correction is an important step to adjust for multiple comparisons. Benjamini-Hochberg or Family wise error (FWE) are common methods. This reduces the likelihood of a false association for each biomarker. Data reduction approaches (e.g., principal components analysis (PCA)) are often applied to reduce the statistical burden of multiple comparisons, given the collinearity between metabolites due to the shared biological pathways. An advantage of data reduction approaches is that the identification of interrelated groups of metabolites can highlight underlying biology in ways that selection of single metabolite analysis cannot. Other commonly used methods to optimize biomarker identification in high-dimensional metabolomic data include partial least squares-discriminant analysis (PLS-DA), least absolute shrinkage and selection operator (LASSO), and rule-based approaches (e.g., random forests). The identified biomarkers, or groups of biomarkers can then be used to build a parsimonious model to identify disease (diagnosis) or predict disease outcomes (prognosis). Newer approaches include the use of higher levels of machine learning to identify important differences between groups [105].

Such multivariate approaches require stringent model validation for the results to be robust and reproducible. Typically, the study population is randomly divided into two groups, a training set and a validation set. The training set is used to build the model and identify the most predictive biomarkers. The validation set is used to determine whether the validity of the biomarkers/model is maintained. Cross-validation is usually performed to test the biomarkers/model in multiple mutually exclusive training and validation sets and is meant to compensate for overfitting. In the best studies, there is also a third, independently collected and analyzed dataset, the test set. The test set is meant to further validate the robustness of the model on new data and is considered gold standard for a study. The predictive performance can be evaluated and compared in each data set using discrimination statistics (sensitivity, specificity), using Harrell’s c-statistic [106] or plotted on a receiver operative characteristic (ROC) curve. To measure risk reclassification after adding the new biomarkers to a base model (e.g., incremental predictive value), both net reclassification indexes as well as integrated discrimination improvement can be calculated [107].

Following biomarker identification, external validation is essential in order to further establish a biomarker’s performance and evaluate whether it can be generalized to other populations other than the one used during development. Although internal validation is also meant to establish a biomarker’s performance, it involves collecting, processing, and analyzing samples under identical conditions, which could naturally carry biases between the training and validation sets. Therefore, validation of the identified biomarkers in large external cohorts which closely represent the discovery cohort should be carried out. Following adequate validation, the next steps toward commercialization of the biomarker(s) can be considered.

In our review we found that most metabolomic studies employed their own combination of statistical models specific to their data, but some overlap can be noted. In n = 24 studies multiple hypothesis testing was used to reduce the false discovery rate (FDR). Some studies which did not perform multiple hypothesis testing were focused on validating a previously discovered biomarker/model [17,26,50]. There were n = 21 studies which used a dimensional reduction with the most common being PCA (n = 12 studies), followed by LASSO (n = 6). Cox Regression was the most common method for evaluating the association of a model with the outcome (n = 33). In n = 34 studies, a prediction model with discrimination statistics was reported but only n = 15 were externally validated. Of the validated biomarkers/models, the most common lipid metabolite class included in the final model was phospholipids (n = 30 biomarkers across 10 studies), followed by sphingolipids (n = 18 biomarkers across 9 studies). The most common outcome of the externally validated studies was predicting incident heart failure (n = 6) as well as incident composite CVD (n = 6).

### 2.5. Diagnostic and Prognostic Value

Currently, the clinical standard for diagnosis and prognosis for CVD depend on protein-based biomarkers such as troponins and N-terminal pro-B-type natriuretic peptide (NT-proBNP) for diagnoses, and LDL or HDL for prognosis. Other protein and hormonal biomarkers have been investigated [108,109,110]; however, these biomarkers might not be as specific as metabolomic biomarkers. Unlike these conventional clinical analytes, metabolomic biomarkers are heavily influenced by demographic, nutritional, medication, and environmental factors which may provide a more precise phenotype and add diagnostic and/or prognostic value.

The added predictive value of different biomarkers was assessed in n = 31 studies which reported discrimination statistics (c-statistic/AUC). Overall, the average increase in c-statistics/AUC after adding metabolite biomarker to a base model was 0.0549 (SE 0.0141). The most common class of metabolite biomarkers used in the prediction models was phospholipids (n = 53 biomarkers across 19 models), followed by sphingolipids (n = 35 across 15 models). For congestive heart failure (CHF) prediction studies (n = 8), the average increase in c-statistics/AUC was 0.0752 (SE 0.0460), with the most common biomarker class of sphingolipids (n = 10 across 6 models), followed by phospholipids (n = 4 across 4 models). A major challenge with comparing metabolomic studies stems from the variability of techniques and data reporting across studies, but it can be foreseen that more commercialization efforts of metabolomic biomarkers will emerge as the number of validation studies increases.

## 3. Lipid Metabolism Translation from Human CVD Studies

Lipids are essential for the short-term metabolic flexibility of the heart to consistently generate the required energy to adequately function. Decreased fatty acid oxidation and a greater reliance on glycolysis for ATP production is a major metabolic characteristic of the failing heart [111]. The lipidome could represent the integration of information stemming from the heart’s metabolic flexibility and the energy substrate availability. Even though changes in the levels of numerous metabolites have been shown to occur in the failing heart (BCAA, lactate, ketones), specific lipid metabolites appear to consistently change in metabolomic profiles of CVD patients, namely sphingolipids, phospholipids, glycolipids, cholesterol esters, fatty acids and acylcarnitines. Previous systematic reviews and a meta-analysis of metabolomic prediction studies in CVD found the majority of studies reported altered lipid metabolites [112,113].

In lipid metabolism, metabolically closely related compounds can have opposite systemic effects, for example, initiating versus resolving inflammatory responses, likely producing divergent disease associations of correlated metabolites. Therefore, distinction of different metabolite species within a lipid class may be important to distinguish different biological processes. For instance, cell experiments show that ceramide 16:0 is proapoptotic, while ceramide 24:0 seems to be protective against apoptosis [114]. Alsehry et al. and Fernandez et al. identified specific triacylglycerol (TG) species which were associated with a decreased risk of CVD, even though the consensus is total increased plasma TG concentration is considered a risk factor for CVD [12]. Conflicting results of lipid species is also not unusual in human studies. For example, ceramide C24:0 was associated with lower risk of CVD in the Framingham Heart Study and Study of Health in Pomerania [37] but was associated with higher risk of CVD in the PREDIMED Trial [58]. A recent meta-analysis of metabolomic biomarkers in CVD prognosis studies found opposing effect sizes of metabolite biomarkers in all classes except for acylcarnitines [112].

Recently, Tomczyk et al. reviewed lipidomic findings from a variety of CVD models in animal, in vitro, tissue, and human studies, and compared the direction of change of biomarkers. We were not interested in the direction of the change of metabolites in this review since we are not confident that useful information based on direction of change can be extrapolated due to the layers of heterogeneity in human metabolomic studies. For example, some studies include patients with complex syndromes such as type 2 diabetes which involve multiple organ systems that may affect the blood-based metabolomic profile. It is possible that an aberrant metabolite concentration may be due to comorbidities, disease severity, or medication effects that may influence different metabolic pathways and be reflected in the blood profile. Therefore, human studies cannot be considered definitive in informing us about pathomechanisms of CVD. Extrapolating information of defective metabolic mechanisms based on blood samples must be interpreted with caution. Regardless of these limitations, summarizing lipidomic findings in human studies can enhance our understanding of disease pathomechanisms or help guide researchers to discover more useful biomarkers, especially when considering a targeted approach or specific cohorts.

### 3.1. Summary of Lipidomic Findings and Potential Pathomechanisms

From the n = 57 studies, there was a total of n = 298 lipid biomarkers across n = 6 lipid subclasses. The most commonly reported metabolite class was phospholipids with n = 130 biomarkers across n = 30 studies, followed by sphingolipids n = 86 biomarkers in n = 28 studies, glycolipids n = 23 biomarkers in 12 studies, cholesterol esters (CE) n = 19 biomarkers in n = 9 studies, FA n = 30 biomarkers in n = 11 studies, acylcarnitines n = 10 in n = 6 studies. In cohorts of coronary artery disease (CAD) and atherosclerosis (n = 11), phospholipid biomarkers were most commonly reported (n = 59) followed by sphingolipids (n = 40), CE (n = 10), and glycolipids (n = 3). In studies, which measured the association with incident CVD (n = 20), phospholipid biomarkers were most commonly reported (n = 35) followed by sphingolipids (n = 20), and glycolipids (n = 16). In studies of incident CHF (n = 9) sphingolipids biomarkers were most commonly reported (n = 15) followed by phospholipids (n = 10), and FA (n = 7). Possible mechanisms connected to these findings include atherosclerosis, cardiomyocyte apoptosis, inflammation, oxidative stress, and insulin resistance, briefly discussed below.

### 3.2. Acylcarnitines and Fatty Acids

The healthy heart is characterized by metabolic flexibility, that is, the ability to switch between energy sources to adapt to changing physiological, environmental, or dietary conditions, with the primary fuel source of long fatty acids. The failing heart develops a metabolic inflexibility characterized by inefficient β-oxidation of FA and a switch to glucose utilization as the primary energy source [2]. This impaired metabolic flexibility can lead to an accumulation of FA oxidation intermediates such as acylcarnitines. The main function of free carnitine (L-carnitine) is to transport long-chain fatty acids—as acylcarnitines—across the inner mitochondrial membrane, thereby delivering these substrates for ATP production [115,116]. Ahmad et al. found higher levels of long chain acylcarnitines (C16 and C18) in CHF patients, which then decreased with LVAD support [4]. Medium and long chain acylcarnitines were previously found to be associated with CVD events [40,42,43]. Thus, these metabolites may reflect altered mitochondrial fatty acid oxidation in CVD, although additional investigation is required to determine the mechanistic causes of the increased circulating acylcarnitine pool.

Carley et al. recently found an unexpected preference for short chain fatty acids (SCFAs) in the failing heart. SCFAs are products of fiber-rich diet degradation by the gut microbiome. Particularly, butyrate showed a higher affinity in mitochondrial oxidation than its ketone bodies counterpart [117]. Reduced butyrate production was previously found in HF patients [118], while high levels of valerate have been correlated with CAD events [19].

Myocardial fatty acid oxidation rates in the failing heart is still a controversial topic, with some studies reporting an increase, decrease, or no change in fatty acid metabolism [119]. Polyunsaturated fatty acids (PUFAs) and their subclasses are the main type of fatty acids of interest in CVD metabolomic studies. Long-chain n-3 PUFAs may have antiatherogenic effects and improve endothelial function as observed in experimental and epidemiological studies [120,121,122]. Würtz et al. observed higher concentrations of omega-6 FA, total PUFA’s, and docosahexaenoic acid (DHA; an omega-3 fatty acid) were associated with lower CVD risk [60]. Results from other cohort studies suggest that PUFAs are associated with a lower CVD risk [123], and dietary consumption of PUFAs can lower cardiovascular risk [124,125], but intervention trials do not suggest risk reduction by PUFA supplementation [126,127]. The role of PUFAs in nutritional supplementation as well as other micro and macronutrients have been previously reviewed [128], although controversy of the role of PUFAs and fatty acid oxidation in CVD pathophysiology remains.

### 3.3. Phospholipids

Phospholipid species may represent biomarkers for the early detection of heightened oxidative stress associated with CVD. Inefficient ATP production can lead to increased reactive oxidative species (ROS) generation and further oxidation of phospholipids [129,130]. Oxidative stress can lead to myocardium impairment (e.g., hypoxia), accelerating the progression of cardiovascular diseases [131]. Oxidation of phospholipids and cholesterol in LDL plays an important role in the progression of atherosclerosis [132], and Lu et al. found that oxidized phospholipids, were significantly elevated in plasma of MI patients [24]. Phospholipids are also important in maintaining HDL integrity and stability and prevent HDL clearance from plasma [133]. In a recent review of lipidomic lipoprotein studies in CVD, Ding et al. showed only the associations of phospholipids with CVD outcomes remained after adjusting for HDL-c and LDL-c [134]. This indicates that phospholipids may be a valuable biomarker independent of total cholesterol and HDL-c.

Phospholipid molecules are shown to be increased and decreased in different models of CVD. The opposing sensitivity of different PC species to future cardiovascular events may relate to the instability of the PC species under heightened oxidative stress or the altered HDL composition and impaired function associated with CVD [135,136]. In general, it has been observed that PC species containing long chain saturated and monounsaturated FAs positively associate with mortality, while PC with long-chain PUFAs appeared to be associated with a protective effect. One of the most studied phospholipid-related metabolites is trimethylamine-N-oxide (TMAO), which is generated in the liver through the oxidation of trimethylamine (TMA). TMA is produced by the gut microbiota in the intestinal tract through a pathway involving dietary nutrients such as phosphatidylcholine, choline, and carnitine [137]. High levels of TMAO are known to promote atherosclerosis and thrombosis [138]. The link between TMAO and cardiovascular risk in humans was first reported by Wang et al. 2011 [57] and further validated in different populations such as CHF and CAD among others [50,51,139,140,141,142,143].

### 3.4. Glycolipids

Glycolipids comprises the bulk of storage fat in tissues. Esterification of one, two or three fatty acyls to glycerol lead to the formation of monoacylglycerol (MG), diacylglycerol (DG) and TG species. MGs and DGs represent intermediates in the biosynthesis and hydrolysis of TGs and function as second messengers in signal transduction pathways such as insulin-signaling pathway [144,145,146,147]. The molecules of TG suppress insulin receptors, thus inducing peripheral insulin resistance [146,147]. DG accumulation has been linked to impaired insulin-stimulated glucose oxidation in the heart [148] as well as insulin resistance and mitochondrial dysfunction [149]. It has previously been shown that the breakdown and synthesis of triglycerides by DG and MG have a causal effect on CVD risk [150].

The relationship between total TGs and CVD risk is well established; however, the relationships between individual glycolipid species and CVD are not. Studies of individual TGs may help better characterize insulin resistance and CVD better than total TGs. For instance, Ganna et al. found that adding MG 18:2 to a model with main cardiovascular risk factors was a better predictor of CVD than total TGs [14]. As seen with other metabolite classes and due to the heterogeneity of the glycolipid class, different species can have opposing associations to CVD risk. It has been shown that saturated TG 16:0 fatty acid was positively associated with fasting serum insulin concentrations and but unsaturated TG 18:3 was negatively associated [146]. Kotronen et al. found that saturated and monosaturated TG molecules (TG (16:0/16:0/18:1) and TG (16:0/18:1/18:0)) correlated positively with insulin resistance, whereas those containing essential fatty acids, such as TG (18:1/18:2/18:2), correlated negatively [147]. Moreover, in the prospective population-based Bruneck study, a specific cluster of TGs with low carbon number and double-bond content, (saturated and monounsaturated) was most consistently associated with CVD [46]. Applying a network-based analytical method, Wang et al. linked a DG and MG cluster to increased CVD risk [56]. These findings may support the link of MGs, DGs, and short saturated TGs to insulin resistance and associated CVD.

### 3.5. Cholesterol Esters

A variety of lipid particles enter and accumulate in the artery wall; however, cholesterol esters and native cholesterol are the most common. Most of the CE enters the artery as components of lipoproteins (LDL, VLDL, HDL) which have been identified in atherosclerotic lesions. The relationship of the vascular matrix, deposits of CE in the arterial wall and its contribution to atherosclerosis is well-documented [151,152,153]. The specific species composition of the CE is likely to be an important atherogenic factor. Infiltrating LDL particles containing a CE-rich core with linoleic acid [CE (18:2)] are thought to be less atherogenic [154]. LDL particles enriched with monounsaturated CE (CE 18:1) are larger and more active in binding to arterial proteoglycans, leading to the subsequent formation of atherosclerotic lesions [155].

Altered levels of CE found in the blood may be due to deficient conversion of free cholesterol to CE (catalyzed by Lecithin-Cholesterol-Acyl-Transferase (LCAT)). In a lipidomic analysis of CVD patients it was found that the ratios of concentrations of CE to free cholesterol were lower in the CVD cohorts than in the control cohort, indicating a deficient conversion of free cholesterol to CE in the blood plasma [156]. In a population-based lipidomic study, it was found that monounsaturated CE (16:1) was the most positively associated with the risk of CVD [46]. In the PREDIMED trial it was found that highly unsaturated CEs were inversely associated with CVD [39,56,58]. A previous prospective cohort study also found CE (16:1) and (CE 18:1) to be positively associated with CAD in acute coronary syndrome patients.

### 3.6. Sphingolipids/Ceramides

Sphingolipids and their precursors ceramides (Cer), may be involved in the pathogenesis of CVD through multiple pathways including inflammation [157], atherosclerosis [158], and apoptosis [159]. In the failing heart, the heart shows remodeling with increased fibrosis in the matrix [160] with myocyte loss by apoptosis occurring in parallel with the onset of fibrosis [161,162]. Increased ceramide species in plasma have been associated with increased cardiac remodeling and cardiac dysfunction in humans [33]. The specific ceramides Cer (d18:1/16:0), Cer (d18:1/18:0), and Cer (d18:1/24:1) were consistently found to be associated with CVD outcomes in the FINRISK study [16], the Corogene study [22], and the PREDIMED trial [58]. In the LURIC study, three sphingomyelin species were associated with mortality [44]. The positive association between ceramide and risk in CVD may also be due to the influence of ceramide on the function of LDLs, since ceramides are primarily contained in LDLs. A previous study has shown that LDLs extracted from human atherosclerosis lesions are highly enriched in ceramides [163].

Although, the biochemical pathways responsible for altered sphingolipid synthesis and metabolism in CVD still incompletely understood, they have shown the best prognostic value out of the other metabolite classes thus far. For instance, the ceramide score CERT1 was originally developed for CAD patients by Zora Biosciences and validated in multiple prospective clinical studies. It has since been updated to CERT2 [7,16,17,22,164]. Another sphingolipid-based score for CAD patients named the sphingolipid-inclusive CAD (SIC) risk score outperformed the CERT1 score and conventional CVD biomarkers in an exploratory analysis. The only metabolomics-based score recommended in the clinic is a ceramide-based score for the prediction of CVD events are in patients with established CAD (https://news.mayocliniclabs.com/ceramides-miheart/. Accessed 5 June 2021). Thus, through lipidomics studies, plasma ceramides have emerged as the most promising new metabolite-based biomarker for CVD with clinical application.

## 4. Future Directions

Early detection and prevention of CVD could help reduce the socio-economic, psychological, and physical burdens on patients. Several factors like the diversity of CVD pathology and its preceding metabolic events make early CVD detection and prevention challenging. Traditional lipid profiling that measures HDL-C, LDL-C, triglycerides and total cholesterol, do not reflect precise molecular perturbations in lipid metabolism associated with CVD onset and progression. Thus, early-stage or long-term prediction scores incorporating metabolomic and traditional biomarkers are emerging as powerful tools in CVD risk management. In addition, short-term prognostic biomarkers or scores of hospitalized or acute care patients can help clinicians make more informed decisions. The ability to detect blood or urine metabolites in near real-time could allow clinicians to monitor worsening or improving clinical trajectories and to target early interventions. Monitoring changes in metabolite profiles over time, with aging, pre/post-surgery, or following medication administration could be used to define an individual’s predisposition for disease or response to therapy. Identification of novel drug targets and customization of drug dosing are also emerging applications of metabolomic technology [165,166].

In parallel and in support of future clinical applications is the ability of metabolomics to help uncover biological mechanisms. Multiomic or panomic approaches integrate multiple “omes”, such as the genome, proteome, transcriptome, epigenome and microbiome and can provide more precise maps of physiological networks of CVD. For example, the CardioNet study used this approach to map the metabolic network of human cardiomyocytes and has the ability to model the flux rates under various conditions [167]. A major challenge of multi-omics and systems biology approaches is the ability to integrate and manage large diverse molecular datasets which require more sophisticated computational and statistical approaches. As technology and methodologies evolve, the molecular complexity of cardiovascular and other diseases will become more and more illuminated while contributing to novel diagnostic, prognostic, or therapeutic strategies.

The future translation of metabolomics to the clinical setting will require significant investment in infrastructure, protocol standardization, education of providers and patients, regulatory and reimbursement structure [96]. Most importantly, metabolomic analyte quantitation needs to first be standardized and then developed to clinical laboratory standards. As throughput technologies have become more powerful and databases for compound identification more robust, standardized procedures are increasingly being applied to large clinical cohorts and we are beginning to see consistency in the findings reported across different metabolomics studies. Ultimately, the goal is to efficiently and effectively conduct molecular phenotyping to advance the goals of precision medicine, thus analysis of biomarkers and mechanisms such as lipid metabolites in CVD is a step toward that direction.

## 5. Conclusions

Our review shows that lipid-based metabolite biomarkers can assist in the diagnosis and prognosis of CVD, but caution must be applied in how we infer the associated peripheral metabolic perturbations. Lipidomic results from human studies have not, thus far, been able to provide detailed and consistent information on the underlying pathomechanisms of CVD. Our review found conflicting results of changes of individual metabolite species across studies, possibly due to high levels of heterogeneity in regard to study design and analytical platforms/approaches. Therefore, our knowledge at the biological level of CVD in humans is mostly related to the classes of lipids (acylcarnitines/fatty acids, phospholipids, glycolipids, cholesterol esters, sphingolipids/ceramides) and their associations with outcomes, rather than single lipid species. The heterogeneity of the study designs and analyses make comparison of results challenging and represent an opportunity for more standardization in this emerging field.

## Figures and Tables

**Table 1:** Study characteristics of lipidomic studies in cardiovascular disease.

First Author, Year	Study Design	Sample Matrix	Platform	Targeted vs. Untargeted	Outcome	Cohort Characteristics	Candidate Lipid or Lipid-Related Biomarkers
Ahmad, 2016 [4]	Case-cohort	Plasma	FIA-MS/MS	Not Specified	CHF Death/Event	CHF patients; 29% female; 64 mean age; 67% white	Long chain acylcarnitines
Alshehry, 2016 [5]	Case-cohort	Plasma	LC-MS/MS	Targeted	Incident CVD in T2DM	2 cohorts of T2DM patients; 39% female; 67 mean age; 20 countries from Asia, Australasia, Europe, and North America	PC(O-36:1), CE(18:0), PE(O-36:4), PC(28:0), LPC(20:0), PC(35:4), LPC(18:2), DG(16:0_22:5), SM (34:1), PC (O-36:5)
Andersson, 2020 [6]	Cohort, prospective	Plasma	LC-MS/MS	Targeted	Incident HF	Community-based cohort; 53% female; 55 mean age; MA, USA cohort	PC 36:4, LPC 18:2
Anroedh, 2018 [7]	Case-cohort	Plasma	LC-MS/MS, FIA-MS/MS	Targeted	CVD event/death	Patients who underwent diagnostic CAG or PCI for ACS or stable angina pectoris; 25% female; 62 mean age; Netherlands medical center	Cer(d18:1/16:0), Cer(d18:1/20:0), Cer(d18:1/24:1), Cer(d18:1/24:0)
Cavus, 2019 [8]	Case-cohort	Serum	LC-MS/MS, FIA-MS/MS	Targeted	Incident CHD	Population-based cohort; 39% female; 57 mean age; 6 European cohorts: Finland, 2 Italy cohorts, Germany, Denmark, Scotland	acyl-alkyl-PC C40:6, diacyl-PC C40:6, acyl-alkyl-PC C38:6, diacyl-PC C38:6, and diacyl-PC
Cheng, 2015 [9]	Case-control	Plasma	LC-MS/MS, FIA-MS/MS	Untargeted and Targeted	CHF Diagnosis	CHF patients; 27% female; 61 mean age; Taiwan medical center	PC C34:4
Cheng-Laaksonen, 2015 [10]	Case-cohort	Plasma	LC-MS/MS	Targeted	CVD event/death	Patients who underwent diagnostic CAG or PCI for ACS or stable angina pectoris; 25% female; 62 mean age; Netherlands medical center	Cer-d18:1/16:0
Delles, 2018 [11]	Case-cohort	Serum	NMR	Targeted	Incident HF hospitalization	Elderly individuals at high risk of CVD; 52% female; 77 mean age; 1 Scotland, 1 Ireland, 1 Netherlands cohort	SCFA (acetate), phenylalanine
Fernandez, 2013 [12]	Case-control	Plasma	FIA-MS/MS	Targeted	Incident CVD	Population-based cohort; 47% female; 60 mean age; Swedish cohort	LPC16:0, LPC20:4, SM 38:2, TG48:1, TG48:2, TG48:3, TG50:3, TG50:4
Floegel, 2018 [13]	Cohort, prospective	Plasma	LC-MS/MS, FIA-MS/MS	Targeted	Incident MI	2 Population-based cohorts; 61% female; 49 mean age; 2 German cohorts	Acylalkyl-PC (C36:3), diacyl-PC (C38:3 and C40:4)
Ganna, 2014 [14]	Cohort, prospective	Plasma	LC-MS/MS	Untargeted	Incident CVD	3 Population-based cohorts; 37% female; 69 mean age; Northern European	LPC-18:1, LPC-18:2, MG (18:2), and SM-28:1
Gao, 2017 [15]	Case-control	Plasma	LC-MS/MS	Untargeted	Incident CAD	Patients undergoing diagnostic CAG; 49% female; 59 mean age; Chinese medical center	LPC (20:4), LPC (16:0), PG(18:0/0:0), elaidic acid, MG (0:0/18:2(9Z,12Z)/0:0), DG (20:2(11Z,14Z)/18:3(9Z,12Z,15Z)/0:0)
Havulinna, 2016 [16]	Cohort, prospective	Serum	LC-MS/MS	Targeted	Incident CVD	Population-based cohort; 53% female; 49 mean age; Finnish cohort	Cer-d18:1/18:0
Hilvo, 2020 [17]	Cohort, prospective	Plasma and Serum	LC-MS/MS	Targeted	CVD event/death	3 CHD cohorts; 21% female; 65 mean age; 1 Norwegian, 1 German, 1 Australian cohort	Cer(d18:1/16:0), Cer(d18:1/18:0), Cer(d18:1/24:1), Cer(d18:1/24:0), PC(16:0/16:0), PC(16:0/22:5), PC(14:0/22:6)
Holmes, 2018 [18]	Nested case-control	Plasma	NMR	Targeted	Incident CVD	Population-based cohort; 52% female; 45 mean age; Chinese cohort	Total FA, omega-6 FA, linoleic acid, PUFA
Jadoon, 2018 [19]	Case-cohort	Serum	LC-MS/MS	Targeted	CKD + Incident CVD	CKD patients; 49% female; 62 mean age; 70% white	SCFA (valerate)
Ji, 2018 [20]	Case-control	Serum	LC-MS/MS	Targeted	CHF progression	CHF patients; 20% female; 57 mean age; NY, USA medical center	Cer16, Cer18, Cer20:1, Cer20, Cer22:1, and Cer24:1
Kalim, 2013 [21]	Nested case-control	Plasma	LC-MS/MS	Targeted	CVD death	Hemodialysis patients; 47% female; 70 mean age; 69% white	Oleoylcarnitine (C18:1)
Laaksonen, 2016 [22]	Case-cohort	Plasma	LC-MS/MS	Targeted	CVD death	Patients undergoing CAG; 31% female; 69 mean age; Finnish, Norwegian, and Swiss cohorts	Cer(d18:1/16:0), Cer(d18:1/24:1), Cer(d18:1/16:0)/Cer(d18:1/24:0), Cer(d18:1/18:0)/Cer(d18:1/24:0), Cer(d18:1/24:1)/Cer(d18:1/24:0)
Lemaitre, 2019 [23]	Cohort, prospective	Plasma	LC-MS/MS	Targeted	Incident HF	Population-based cohort; 60% female; 76 mean age; 16% black from 4 US communities NC, CA, MD, PA	Cer-16, SM-16, Cer-22, SM-20, SM-22, and SM-24
Lu, 2017 [24]	Case-control	Plasma	LC-MS	Untargeted and Targeted	MI	MI and stable angina patients; 75% female; 59 mean age; China medical center	9 oxyphospholipids (HODA-PC, KDdiA-PC, D2/E2-IsoP-PC, PEIPC, HETE-PC, IsoF-PC, PECPC, F2-IsoP-PC, HODE-PC), 9 hydrolyzed FA (20-HETE, 11,12 DHET, 13-HODE, 5-HETE, D2/E2-IsoP, 14,15-DHET, 5,6-DHET, 14(15)-EET, 9-HODE)
Mayerhofer, 2020 [25]	Case-control	Plasma	LC-MS/MS, GC-MS	Targeted	All-cause mortality or listing for heart transplant	CHF patients; 59% female; 59 median age; Norway cohort	TMAO, SCFA (butyrate)
McGranaghan, 2020, 2021 [26,27]	Case-cohort	Serum	LC-MS/MS, GC-MS	Untargeted and Targeted	CHF Death	CHF patients; 26% female; 72 mean age; German medical center	SM d18:1/23:1, SM d18:2/23:0, SM d17:1/24:1, TG 18:1/18:0/18:0, PC 16:0/18:2
Meikle, 2011 [28]	Cross-sectional	Plasma	LC-MS/MS	Targeted	unstable CAD/stable CAD	de Novo CAD patients; 22% female; 66 mean age; Australian cohort	10 species of PE(O)
Miller, 2012 [29]	Cohort, prospective	Plasma	LC-MS/MS	Not Specified	Incident CAD	Chest pain or angina patients; 38% female; 48 mean age; 72% white	CE 16:1, CE 18:1
Mueller-Hennessen, 2017 [30]	Cohort, prospective	Plasma	LC-MS/MS, GC-MS	Untargeted and Targeted	Incident HF	CHF patients; 30% female; 59 mean age; 3 German medical centers	SM d18:1/23:1, SM d18:2/23:0, SM d17:1/24:1, TG 18:1/18:0/18:0, PC 16:0/18:2
Mueller-Hennessen, 2017 [31]	Case-control	Plasma	LC-MS/MS, GC-MS	Untargeted and Targeted	CHF Diagnosis	CHF patients; 0% female; 50 mean age; Germany medical center	Cholesterol, Behenic acid (C22:0), Lignoceric acid (C24:0), Linoleic acid (C18:cis [9,12] 2), Tricosanoic acid (C23:0), LPC (C17:0), LPC (C18:0), LPC (C18:1), LPC (C18:2), PC (C16:1, C18:2), 5-O-Methylsphingosine, erythro-Sphingosine, Phytosphingosine
Mundra, 2018 [32]	Case-cohort	Plasma	LC-MS/MS	Targeted	CVD event/death	Patients with MI or unstable angina; 18% female; 63 median age; Australia and New Zealand medical centers	PC (O-34:2), PC (38:5), PI (38:3), PC (O-36:1), GM3(d18:1/16:0), PI (18:2/0:0), PE (38:6)
Nwabuo, 2019 [33]	Cross-sectional	Plasma	LC-MS/MS	Targeted	Echo measures correlation	Community-based cohort; 65% female; 66 mean age; MA, USA community	Cer16:0/Cer24:0
Ottosson, 2021 [34]	Case-control	Plasma	FIA-MS/MS	Untargeted	Incident CAD	Population-based cohort; 60% female; 58 mean age; Swedish cohort	PC 15:0;0_18:2;0, PC 17:0;0_20:3;0, PC 16:0;0_20:1;0, PC O 16:2;0_18:0;0, SM 34:1;2, DAG 18:1;0_18:3;0, PI 16:0;0_20:4;0; CE 18:0;0
Paapstel, 2017 [35]	Case-control	Serum	LC-MS/MS, FIA-MS/MS	Targeted	Atherosclerosis	PAD and CAD patients; 0% female; 63 mean age; Estonia medical center	PC-diacyl-28:1, PC-diacyl-30:0, PC-diacyl-32:2, PC-acyl-alkyl-30:0, PC-acyl-alkyl-34:2, LPC-acyl-18:2
Paynter, 2018 [36]	Case-control	Plasma	LC-MS, LC-MS/MS	Untargeted	Incident CVD	Post-menopausal women cohort; 100% female; 67 mean age; 77% white	Hydroxy-PC (C34:2)
Peterson, 2018 [37]	Case-control	Plasma	LC-MS/MS	Targeted	Incident CVD; HF	2 Community-based cohorts; 53% female; 60 mean age; 2 US communities MO and MA	C24:0/C16:0
Poss, 2020 [38]	Case-control	Serum	LC-MS/MS	Targeted	Incident CAD	CAD patients; 34% female; 55 mean age; UT, USA medical center	dihydro-cer(d18:0/18:0), cer(d18:1/18:0), cer(d18:1/22:0), cer(d18:1/24:0), dihydro-SM(d18:0/24:1), SM(d18:1/24:0), SM(d18:1/18:0), and sphingosine
Razquin, 2017 [39]	Case-cohort	Plasma	LC-MS	Untargeted	Incident CVD	Population-based cohort; 57% female; 67 mean age; Spanish cohort	Polyunsaturated PCs, LPCs, PC-plasmalogens, CEs, long TGs, short TGs (saturated/monounsaturated), hPCs and, MGs, DGs and PEs
Rizza, 2014 [40]	Cohort, prospective	Serum	LC-MS/MS, FIA-MS/MS	Targeted	CVD event/death	Geriatric ambulatory patients; 43% female; 77 mean age; Italian medical center	medium-long-chain acylcarnitines (acetyl carnitine C2, C6, C8, C10, C10:1, C12, C12:1, C14, C14:1, C14:2, C16, C16:1, C18:1, C18:2)
Seah, 2020 [41]	Cohort, prospective	Plasma	LC-MS/MS	Targeted	CVD event/death	Population-based cohort; 53% female; 49 mean age; Singapore Chinese cohort	total monohexoylceramides, total long-chain sphingolipids (C16–C18), and total 18:1 sphingolipids
Shah, 2010 [42]	Cohort, prospective repository	Plasma	LC-MS/MS	Targeted	CVD event/death	Cardiac catheterization patients; 24% female; 46 mean age; 67% white	Short-chain dicarboxylacylcarnitines; medium-chain acylcarnitines
Shah, 2012 [43]	Cohort, prospective	Plasma	LC-MS/MS	Targeted	All-cause mortality or MI	Cardiac catheterization patients; 38% female; 62 median age; 73% white	Short-chain dicarboxylacylcarnitines, Long-chain dicarboxylacylcarnitines, Fatty acids
Sigruener, 2014 [44]	Cohort, prospective	Plasma	FIA-MS/MS	Targeted	Mortality	Hospitalized coronary angiography patients; 30% female; 63 mean age; 100% white	PC-32:0, SM-16:0, SM-24:1 and CM-24:1
Stegemann, 2011 [45]	Case-control	Plaque; Plasma	FIA-MS/MS	Targeted	Atherosclerosis	Endarterectomy patients; 29% female; 69 mean age; British cohort	10 CEs, 9 SMs, 8 LPCs, and 31 PCs
Stegemann, 2014 [46]	Cohort, prospective	Plasma	FIA-MS/MS	Targeted	Incident CVD	Population-based cohort; 52% female; 66 mean age; 100% white	TG-54:2, CE-16:1, and PC-36:5
Stenemo, 2019 [47]	Cohort, observational	Plasma and Serum	LC-MS/MS	Untargeted	Incident HF	3 Community-based cohorts; 33% female; 70 mean age; 3 Sweden cohorts	SM (30:1)
Sun, 2016 [48]	Nested case-control, prospective	Plasma	GC-MS/MS	Targeted	Incident MI	Population-based cohort; 35% female; 66 mean age; Singapore Chinese cohort	Long-chain n-3 fatty acids, stearic acid, and arachidonic acid
Syme, 2016 [49]	Cohort, observational	Serum	LC-MS/MS	Untargeted	Incident CVD	Population-based cohort; 52% female; 15 median age; Canadian Cohort	PC-16:0/2:0, PC-14:1/0:0
Tang, 2013 [50]	Cohort, prospective	Plasma	LC-MS/MS	Targeted	CVD event/death	Cardiac catheterization patients; 36% female; 63 mean age; Cleveland, Ohio USA Medical Center	TMAO
Tang, 2014 [51]	Cohort, prospective	Plasma	LC-MS/MS	Targeted	All-cause mortality IN CHF	Patients who underwent diagnostic CAG; 41% female; 66 mean age; Cleveland, Ohio USA Medical Center	TMAO
Tarasov, 2014 [52]	Case-control	Serum	LC-MS/MS, FIA-MS/MS	Targeted	CVD Death	CAD patients; 0% female; 66 mean age; German medical center	Cer(d18:1/16:0)/Cer(d18:1/24:0), Cer(d18:1/20:0)/Cer(d18:1/24:0), Cer(d18:1/24:0)/Cer(d18:1/24:1)
Tzoulaki, 2019 [53]	Cohort, prospective	Serum	NMR	Untargeted	Atherosclerosis/Incident CVD	3 Population-based cohorts; 47% female; 63 mean age; 53% white	Triglycerides, Phospholipids, CE
Vaarhorst, 2014 [54]	Case-cohort, prospective	Plasma	NMR	Untargeted	Incident CVD	Population-based cohort; 51% female; 49 mean age; Netherlands cohort	TMAO, an unsaturated lipid structure
Vorkas, 2015 [55]	Cross-sectional	Serum	LC-MS/MS	Untargeted	Calcific CAD	Exertional angina patients; 59% female; 65 mean age; Sweden medical center	PC(16:0/20:4), lysoPC(20:4), PI(18:2/18:0), SM(d17:1/16:0), SM(d18:1/16:0), SM(d17:1/22:0), SM(d18:1/23:0), SM(d18:2/16:0), SM(d18:2/22:0), SM(d18:2/24:1), TG(16:0/18:1/22:5), TG(18:1/18:1/20:4), TG(16:0/18:1/18:1)
Wang-Dong, 2018 [56]	Case-cohort	Plasma	LC-MS	Untargeted	Incident CVD	Population-based cohort; 53% female; 69 mean age; Spanish cohort	hPC, DG, MG, highly unsaturated phospholipids, and CE
Wang-Hazen, 2011 [57]	Case-control	Plasma	LC-MS, LC-MS/MS, GC-MS, NMR	Targeted	Incident CVD	Stable non-symptomatic subjects undergoing elective cardiac evaluations; 51% female; 64 mean age; Cleveland, Ohio USA Medical Center	TMAO, choline, betaine
Wang-Hu, 2017 [58]	Case-cohort, prospective	Plasma	LC-MS/MS	Targeted	Incident CVD	Population-based cohort; 57% female; 67 mean age; Spanish cohort	Cer(16:0), Cer(22:0), Cer(24:0), Cer(24:1)
Wittenbecher, 2021 [59]	Nested case-control, prospective	Plasma	LC-MS, FIA-IM-MS/MS	Untargeted and Targeted	Incident HF	2 Population-based cohorts; 56% female; 72 mean age; 1 German and 1 Spanish cohort	PC C16:0/C16:0 and CerC16:0
Würtz, 2015 [60]	Cohort, prospective	Serum	LC-MS/MS, GC-MS, NMR	Untargeted and Targeted	Incident CVD	3 Population-based cohorts; 57% female; 56 mean age; 1 Finnish and 2 UK cohorts	MUFA, omega-6 fatty acid, docosahexaenoic acids
Zordoky, 2015 [61]	Case-control	Plasma	LC-MS/MS, FIA-MS/MS, NMR	Untargeted and Targeted	HFrEF vs HFpEF	CHF patients; 39% female; 65 mean age; Canadian cohort	2-hydroxybutyrate, octadecenoylcarnitine (C18:1), hydroxyprionylcarnitine (C3-OH), SM(C24:1), octanoylcarnitine, and SM(C20:2)

Abbreviations: ACS, acute coronary syndrome; CA, California; CAD, coronary artery disease; CAG, coronary angiogram; CE, cholesterol ester; Cer, ceramide; CHF, congestive heart failure; CVD, cardiovascular disease; DG, diglycerol; FA, fatty acid; FIA, flow injection analysis (used here for shotgun approaches); GC, gas chromatography; HF, heart failure; HFpEF, heart failure with preserved ejection fraction; HFrEF, heart failure with reduced ejection fraction; hPC, hydroxylated phosphatidylcholine; IM, Ion mobility; LC, liquid chromatography; LPC, lysophosphatidylcholine; MA, Massachusetts; MD, Maryland; MG, monoglycerol; MI, myocardial infarction; MS, mass spectrometry; MUFA, monounsaturated fatty acid; NC, North Carolina; NMR, nuclear magnetic resonance; NY, New York; PA, Pennsylvania; PAD, peripheral artery disease; PC, phosphatidylcholine; PCI, percutaneous coronary intervention; PE, phosphatidylethanolamine; PE(O), alkylphosphatidylethanolamine; PG Phosphatidylglycerol; PI, phosphatidylinsitol; SCFA, short-chain fatty acids; SM, sphingomyelin; TG, triacylglycerol; TMAO, trimethylamine-N-oxide; UK, United Kingdom; UT, Utah.
